# Implementation of a Formal Medical Handover in an Acute Mental Health Unit

**DOI:** 10.1192/bjo.2024.375

**Published:** 2024-08-01

**Authors:** Jonathan Hagan, Victoria Lamont, Lauren McCormick

**Affiliations:** Southern Health and Social Care Trust, Craigavon, United Kingdom

## Abstract

**Aims:**

To implement a robust and reliable medical handover process in the Bluestone Unit, Craigavon Area Hospital, Southern Health and Social Care Trust. Following discussion with Junior Medical Staff, Doctors did not feel confident that they were always aware of outstanding physical investigations or any acutely unwell patients on the inpatient wards. This could lead to patient safety issues which we aimed to address using the PDSA model for effective change management.

**Methods:**

A) Twice daily face to face handover was introduced at 09:00 and 16:45 in the Junior Doctor's office, easily accessible to staff. A standardised handover template was already in existence. The outgoing Doctor On-Call overnight would complete this to handover any outstanding tasks. The Doctor carrying the On-Call bleep during the day would then use this template to lead a formal, face to face handover with a team of Junior Doctors covering each of the inpatient wards. This helped to clarify which wards were covered and ensure timely, effective allocation of tasks.

B) A formal, face to face “weekend handover” was introduced at 15:30 in the Conference Room every Friday afternoon.

A survey was sent to twelve Junior Doctors to gather formal feedback both before and after these interventions.

**Results:**

Following these two interventions:

1. 91.67% feel there is now a structured and comprehensive daily handover. 91.67% felt this was not the case before.
2. 63.64% feel there is now always a successfully completed morning handover. 0% felt this was the case before.
3. 83.33% feel there is now always a successfully completed weekend handover. 41.67% felt this was not the case before.
4. 75% feel they are always aware of sick patients when On-Call. 25% felt they were not at all aware before and 66.67% felt they were only occasionally aware before.
5. 91.67% feel they are always aware of outstanding bloods to chase. 8.33% felt always confident before.
6. 83.33% feel they are always aware of outstanding investigations to chase. 16.67% felt always confident before.
7. 90.9% feel they know which wards are covered and uncovered when carrying On-Call bleep. 9.09% felt they knew before.

**Conclusion:**

The implementation of a formal handover process has significantly improved Doctors' awareness of outstanding tasks and ward cover, which is likely to benefit patient safety moving forward. Further action is necessary to improve communication between medical and nursing colleagues regarding physical investigations performed and appropriate, timely follow up.

